# An Efficient Virus-Induced Gene Silencing System for Functional Genomics Research in Walnut (*Juglans regia* L.) Fruits

**DOI:** 10.3389/fpls.2021.661633

**Published:** 2021-06-23

**Authors:** Yifan Wang, Ning Huang, Niu Ye, Lingyu Qiu, Yadong Li, Huiling Ma

**Affiliations:** College of Life Sciences, Northwest A&F University, Yangling, China

**Keywords:** virus-induced gene silencing, tobacco rattle virus, *Juglans regia*, *Juglans regia* polyphenol oxidase genes, browning, gene function analysis

## Abstract

The Persian walnut (*Juglans regia* L.) is a leading source of woody oil in warm temperate regions and has high nutritional and medicinal values. It also provides both tree nuts and woody products. Nevertheless, incomplete characterization of the walnut genetic system limits the walnut gene function analysis. This study used the tobacco rattle virus (TRV) vector to construct an infectious pTRV-*JrPDS* recombinant clone. A co-culture inoculation method utilizing *Agrobacterium* was screened out from four inoculation methods and optimized to set up an efficient virus-induced gene silencing (VIGS) system for *J. regia* fruit. The optimized VIGS-TRV system induced complete photobleaching phenotype on the walnut fruits of four cultivars, and the *JrPDS* transcript levels decreased by up to 88% at 8 days post-inoculation (dpi). While those of browning-related *J. regia* polyphenol oxidase (PPO) genes *JrPPO1* and *JrPPO2* decreased by 67 and 80% at 8 dpi, respectively, accompanied by a significant reduction in fruit browning phenotype. Sodium dodecyl sulfate-polyacrylamide gel electrophoresis screening and Western Blot showed that the PPO protein levels were significantly reduced. Moreover, a model of TRV-mediated VIGS system for inoculating *J. regia* fruit with efficient silence efficiency *via* co-culture was developed. These results indicate that the VIGS-TRV system is an efficient tool for rapid gene function analysis in *J. regia* fruits.

## Introduction

Walnut (*Juglans regia* L.) is a diploid species (2*n* = 32) of tree nuts and woody oils widely cultivated and globally distributed due to its high nutritional and economic importance (Griel et al., 2007). There is a high demand for *J. regia* products due to the rapid increase in walnut consumption. However, inefficient post-harvest processing technologies lead to the devaluation of products and limit the sustainable development of the *J. regia* industry. Relative to dry *J. regia*, fresh walnuts are crispy delicious, and richer in nutrients (Ma et al., 2013; Wang et al., 2015), making them an ideal alternative to the dried *J. regia* products. The modified atmosphere storage (MAP) of the fresh in-hull fruit is effective in the traditional preservation technologies of fresh *J. regia* nuts (Huiling et al., 2012; Wang et al., 2014). However, the MAP only lasts for about 2–3 months (Wang et al., 2017), far from meeting the annual consumer demand. The storage of fresh *J. regia* fruit is still the major challenge in the current walnut industry.

*Juglans regia* hull is rich in phenols (Colaric et al., 2005; Solar et al., 2006), and under aerobic conditions, polyphenol oxidase (PPO) catalyzes the oxidation of phenols to generate quinones which automatically polymerize into polymers to present browning symptom (Matheis and Whitaker, 1984). A black juice is produced by severely browned tissue, which contaminates the inner nut and thus shortens the storage duration (Liu et al., 2014; Boonyaritthongchai and Supapvanich, 2017; Yuan et al., 2019). Previous researchers have applied the antisense gene technology to inhibit the expression of a specific member of the PPO genes, thus bred browning resistant cultivars of both apple and potato (Murata et al., 2001; Chi et al., 2015). The *J. regia* PPO enzyme is encoded by two genes (Escobar et al., 2008; Martínez-García et al., 2016), which leads to the idea of whether the browning resistant cultivars could be generated by inhibiting individually one of them. To confirm and implement this idea, the functional differences between the two PPO genes in fruit browning should be investigated.

Kumagai et al. (1995) indicated that a phytoene desaturase (PDS) cDNA inserted into the tobacco mosaic virus (TMV) genome could silence the PDS gene in tobacco. PDS is a key rate-limiting enzyme in the carotenoid synthesis pathway (Chen et al., 2008; Furubayashi et al., 2014). It is commonly used as a reference gene to evaluate virus-induced gene silencing (VIGS; Bennypaul et al., 2012; Shen et al., 2015; Zhang et al., 2016). PDS gene silencing causes an albinism symptom in the young leaves of carotenoids-pigmented plants (Sun et al., 1998). Ruiz et al. (1998) inserted a PDS gene into the Potato virus X (PVX) genome and infected the potato with the recombinant virus, causing the leaves to show albino symptoms. Therefore, they proposed that PVX use through VIGS could effectively silence endogenous plant genes for functional analysis of unknown genes.

VIGS is a green post-transcriptional gene silencing (PTGS) technique used for gene function analysis (Ratcliff et al., 1997; Burch-Smith et al., 2004; Gupta et al., 2013). Compared with conventional plant gene transformation methods (Weigel et al., 2000; Kaiser et al., 2002), VIGS is desirable because it is highly efficient and specific and does not require genetic transformation or generation (Huang et al., 2012). More than 30 VIGS vectors have been developed for dicotyledons (Gu et al., 2014; Liou et al., 2014). Some of the successfully used VIGS vectors include the Brome mosaic virus (BMV; Ding et al., 2018), cucumber mosaic virus (CMV; Wang R. et al., 2016), barley stripe mosaic virus (BSMV; Scofield et al., 2005), and tobacco rattle virus (TRV; Zhang et al., 2017). TRV is a relatively simple and cost-effective silencing vector. In addition, it induces mild virus symptoms, offers durable and high silencing efficiency, and can simultaneously silence genes in various plant tissue (Zheng et al., 2010; Kumar et al., 2012). However, the application of VIGS-TRV on tree nuts, including walnut, has not been reported.

Firstly, the feasibility of applying the VIGS-TRV system to silence genes in *J. regia* was verified by silencing the endogenous *J. regia* phytoene desaturase gene (*JrPDS*), an easily observable marker gene (Charrier et al., 2019; Naing et al., 2019). Then, a protocol for TRV virus infection on *J. regia* fruits was optimized using the *PDS* gene, after which the optimized protocol was applied to silence *J. regia* polyphenol oxidase genes (*JrPPOs*). Thus, this study aimed to establish a VIGS-TRV system for evaluating the functions of two JrPPO genes on post-harvest browning resistance of the fresh in-hull walnut fruit.

## Materials and Methods

### Plant Materials and Virus Strains

Mature fruits of four *J. regia* cultivars (Xiluo2, Qingxiang, Xifu1, and Xiangling) were harvested from 10-year-old trees in the walnut garden at Weihe Experimental Station (Yangling, China). From 60 to 136 days after flowering (10th of June to the 25th of August) in 2020, fruits were freshly collected each time when needed for this study. Upon reaching the laboratory, the fruits were kept in the dark at 0°C for 12 h to lose field heat. Then their temperature was stabilized at 20°C for 2 h before inoculation. The VIGS-TRV vectors, consisting of vector pTRV1 and pTRV2, were provided by the research group of Professor Zhenhui Gong from the College of Horticulture, Northwest A&F University (Yangling, China). pTRV1 and pTRV2 are two components of the VIGS-TRV. Detailed information for pTRV1 and pTRV2 was described by Liu et al. (2002).

### Virus-Induced Gene Silencing Vector Construction

**Figure 1:**
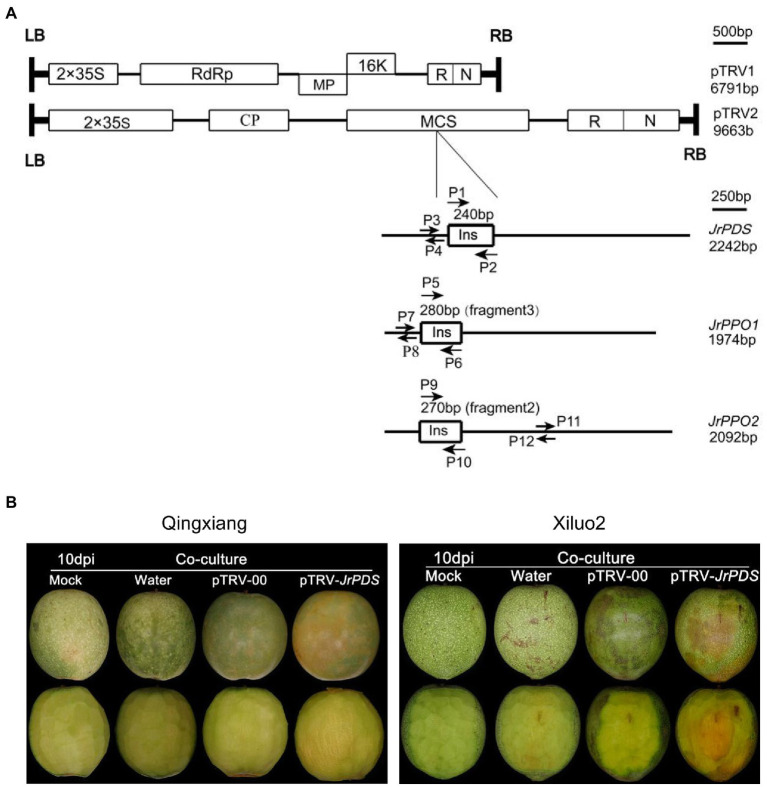
VIGS-TRV system construction and *JrPDS* silencing in *Juglans regia* fruits. **(A)** Genomic organization of the two TRV components, pTRV1 and pTRV2, drawn to a scale of 500 bp. Boxes indicate Open reading frames (ORF). Schematic representation of cDNA full-length for *JrPDS* and *JrPPOs* (black line) drawn to scale of 250 bp. The 240-bp fragment of *JrPDS*, the 280-bp fragment3 of *JrPPO1*, and the 270-bp fragment2 of *JrPPO2* indicated by boxes are inserted in pTRV2. P1 and P2, P5 and P6, P9 and P10 are primer sets for generating pTRV-*JrPDS* and pTRV-*JrPPO* recombinants. P3 and P4, P7 and P8, P11 and P12 are primer sets for qRT-PCR analysis of *JrPDS* and *JrPPOs* transcripts. **(B)** Silencing *JrPDS* in *J. regia* fruits using the pTRV-*JrPDS*. TRV vectors induced photobleaching phenotype at 10 days post-inoculation (dpi), while mild mosaic symptoms appeared on fruits infected with pTRV-00, watery lesions on water control, no changes emerged on mock fruits. TRV, tobacco rattle virus; VIGS, virus-induced gene silencing; and qRT-PCR, quantitative reverse transcription PCR.

Total RNA was extracted from about 1-week-old young walnut leaves using the Plant RNA Kit R6827 (Omega Biotek, United States).^1^ According to the manufacturer’s instructions, the first-strand cDNA was synthesized using an Evo M-MLV RT Kit (AG11707, AG, Changsha, China). The gene sequences of walnut species, including the *JrPDS* gene (GenBank accession number XM_018972693.1), *JrPPO1* gene (GenBank accession number: ACN86310.1), and *JrPPO2* gene (GenBank accession number. XP_018805282.1), were obtained from the NCBI database. All conserved gene sequences for genes *JrPDS*, *JrPPO1*, and *JrPPO2* were obtained from a conservative domain search of the NCBI website.^2^ The nucleic acid sequence aligning to the *J. regia* genome database^3^ showed that the *J. regia PDS* gene family had only one member. Therefore, a 240 bp fragment was randomly selected on its conserved sequences and inserted into the vector pTRV2 (Figure 1A). According to the previous research on apple, the selected JrPPOs fragment was designed from the non-conserved domain (Jiang et al., 2017). The fragments were amplified with primers designed by the software of CE Design (Vazyme, Nanjing, China; Supplementary Table S2).^4^ The pTRV2 vector was then digested with restriction endonuclease *BamH*I and *Xho*I (TAKARA, Japan).^5^ A Gel Extraction Kit D2500 (Omega Biotek)^5^ was used to purify the PCR product and the digested pTRV2 vector. To improve the cloning efficiency, we used the in-Fusion HD Cloning Kit (TAKARA, Japan)^5^ to connect the PCR product with the digested pTRV2 vector. Then, 10 μl of the mixture was transformed into *E. coli* DH5α competent cells (TAKARA).^5^ Transformants were cultured on LB culture medium [1% (w/v) Tryptone, 0.5% (w/v) Yeast Extract, 1% (w/v) NaCl] and tested by PCR amplification using the primers UP-F and UP-R (Supplementary Table S2) with Premix Taq™ (TAKARA).^5^ The PCR procedure was as follows: 30 s at 98°C, followed by 35 cycles of 10 s at 98°C, 30 s at 55°C and 30 s at 72°C. Plasmids from the positive clones were purified and sequenced (Sangon Biotech, Shanghai, China). The selected plasmids were transformed into *Agrobacterium tumefaciens* strain GV3101 for subsequent experiments. The pTRV2 vector without insert was designated pTRV-00, while those with *JrPDS*, *JrPPO1*, and *JrPPO2* were shown as pTRV-JrPDS, pTRV-*JrPPO1*, and pTRV-*JrPPO2*.

### Fruit Incubation and Inoculation Assay

For the trial of VIGS-TRV symptoms on walnut (*J. regia* L.) fruits, two cultivars, “Xiluo” and “Qingxiang,” were employed. Fifty-four fruits were separated randomly for each cultivar in three control inoculations [18 for Mock without treatment, 18 for water, and 18 for pTRV-00 (pTRV1:pTRV2 = 1:1, v/v)]. Four groups of fruits, 18 in each, were also separated for each cultivar to be inoculated with pTRV-*JrPDS* (pTRV1:pTRV-*JrPDS* = 1:1, v/v) by four inoculation methods, respectively, i.e., injection inoculation (Jiang et al., 2017), vacuum infiltration (Maleksaeedi et al., 2014), friction inoculation (Ma et al., 2012), and co-culture (Zheng et al., 2012). The fruits of co-culture treatment were kept in 500 ml tissue culture bottles, three in each, and incubated under 22°C in a shaker at 60 rpm. At the same time, the fruits for the other three inoculation treatments covered with food wrapping film were placed at 22°C according to the method described in each corresponding reference. The fruits were observed every day during the inoculation to record the days when photobleaching on the fruits appeared and the number of fruits that occurred to calculate the infection efficiency for each group. The fruits were immediately photographed after removed from the inoculation solution at 10 and 20 days post-inoculation (dpi), respectively. Each treatment was repeated three times to obtain 54 fruits as the calculation basis of the success rate of inoculation.

Among the above four inoculation methods, co-culture was the most effective (Supplementary Table S1), which specific steps were described as follows. A single colony of *A. tumefaciens* strain GV3101 harboring TRV was cultured in an LB medium (50 mg/L kanamycin and 50 mg/L rifampicin) at 28°C overnight while shaking at 220 rpm. The bacterial cells were harvested *via* centrifugation at 5,000 × *g* for 10 min when the density of the culture reached an OD_600_ of 0.6. The OD_600_ was then adjusted to 0.1 in a transformation solution containing 1/2 MS, 100 μM acetosyringone (AS), 75 μM Triton X-100, and 2.5% (w/v) sucrose (pH 5.8) for plant co-culture research. Before *J. regia* fruits and *A. tumefaciens* were co-cultivated, the fruits were wounded at four sites on each fruit. They put in 1/2 MS solution (pH 5.8) with 25% (w/v) sucrose for 5 h hyperosmotic pretreatment to improve pathogen inoculation efficacy. After that, the *J. regia* fruits were put in a tissue culture bottle with the configured transformation solution and incubated in a shaker at 60 rpm (16 h light at 22°C/8 h dark at 18°C and 60% humidity). The solution was refreshed every 24 h.

To optimize the co-culture parameters of VIGS-TRV, the fruits of *J. regia* cv. Qingxiang, Xiluo2, Xifu1, and Xiangling were inoculated by the co-culture method at different conditions. First, the inoculated fruits were photographed after washing at 10 dpi. Then, the hull of walnut fruit was sampled quickly by cutting off 1/4 of the hull of each fruit with a clean surgical blade. The sampled hulls were cut into particles of about 2–3 mm in diameter, mixed, and immediately frozen in liquid nitrogen and stored at −80°C until use. The days when the photobleaching phenotype appeared were recorded, and infection efficiency was calculated in the same way as described above. This experiment was performed with three independent biological replicates, and 18 fruits were included in each replication per cultivar.

The fruits of cv. Qingxiang and Xiluo2 were used to further optimize the VIGS-TRV protocol by silencing *JrPDS*. The fruits of each treatment were photographed and sampled at a 2-day interval with the same sampling method above. This experiment was performed with three groups (repeats) of fruits from different trees, and 18 fruits were included in each replication per cultivar.

### *JrPPOs*-Silencing Fragment Screening

*Juglans regia* polyphenol oxidase genes are major relevant factors to the browning of *J. regia* fruit. They are thus selected as the target genes to test the VIGS-TRV system established. Since the conserved domains of the two JrPPO genes are identical, if the conserved domains are chosen to create a silent segment, the *JrPPO1* and *JrPPO2* will be silenced simultaneously. Therefore, the fragments located in the non-conserved sequences of each gene should be selected to discern the function of the *JrPPO1* and the *JrPPO2* (Jiang et al., 2017). Three fragments per gene were chosen randomly with approximately 300 bp in length. Each fragment was amplified (Figure 1A; Supplementary Table S2) and inserted into the pTRV2 vector. A total of 126 fruits were randomly divided into seven groups, 18 in each, to accept different infections: control (pTRV-00; pTRV1:pTRV-00 = 1:1, v/v); pTRV-Fragment1-, pTRV-Fragment2- and pTRV-Fragment3- *JrPPO1*; pTRV-Fragment1-, pTRV-Fragment2-, and pTRV-Fragment3-*JrPPO2.* Each inoculation solution was the mixture (pTRV1:pTRV-Fragmentx = 1:1, v/v, *x* = 1,2,3). At 8 dpi, the fruits of each treatment were removed from the inoculation solution for 1 h. At this time, they showed obvious differences in phenotype. All independent experiments were performed in “QingXiang” fruits with three biological replicates each. The fragment with the highest silence efficiency obtained from reverse transcription PCR (RT-PCR) and quantitative reverse transcription PCR (qRT-PCR) was screened as the most effective silencing fragment for each JrPPO gene.

### Reverse Transcription PCR and Quantitative Reverse Transcription PCR

Plant RNA Kit R6827 (Omega Biotek)^1^ was used to extract total RNA from the frozen sample of the walnut hull. An Evo M-MLV RT Kit (AG11707, Accurate Biology)^6^ for RT-PCR and qRT-PCR was used following the instructions of the manufacturer to synthesize the first-strand cDNA. RT-PCR analysis was performed as described by Liu et al. (2002). An oligo (dT) primer was used to reverse-transcribe 1 μg total RNA, and the specific primer sequences (Supplementary Table S3) were used to assay the expression of each gene using RT-PCR or qRT-PCR. An Applied Biosystems 7900HT Fast Real-Time PCR system (Life Technologies)^7^ with fourfold diluted walnut cDNA and an SYBR® Green Premix Pro Taq HS qPCR Kit (AG11701, Accurate Biology)^6^ was used for qRT-PCR analysis. The specific primers (Supplementary Table S3) were used for *JrGAPDH* (internal reference gene) mRNA level determination to normalize transcript levels in the samples. The relative gene expression levels were calculated using the 2^−ΔΔCT^ method (Livak and Schmittgen, 2001). The Student’s *t*-test was used to determine the differences between the treatments. Three technical replications and three independent biological replications were included for each experiment.

### Polyphenol Oxidase Activity Assay and SDS–PAGE Analysis

A total of 72 fruits were randomly divided into four groups; 18 control fruits (pTRV-00; pTRV1:pTRV-00 = 1:1, v/v), 18 pTRV-*JrPPO1*- [pTRV1:pTRV-Fragment3 (*JrPPO1*) = 1:1, v/v], 18 pTRV-*JrPPO2*- [pTRV1:pTRV-Fragment2 (*JrPPO2*) = 1:1, v/v], and 18 pTRV-*JrPPOs*-treated fruits (pTRV1:pTRV-*JrPPO1*:pTRV-*JrPPO2* = 2:1:1, v/v/v). The treated fruits were picked at 8 dpi for photographing and sampling with the same method as described in the part of inoculation assay. All independent experiments were performed in “Qingxiang” fruits with three biological replicates each.

About 0.5 g frozen *J. regia* hull samples (“Qingxiang”) were ground in 50 mM phosphate buffer (pH 6.8, 4°C pre-chilled) and volumed up to 6 ml. The mixture was centrifuged at 10,000 × *g*, 4°C, for 20 min to obtain a crude PPO enzyme solution (Wang J. et al., 2016). The Amicon® Ultra-0.5 Centrifugal Filter Devices (Merckmillipore)^8^ were used to concentrate the protein solution. A Nanodrop ND-1000 spectrophotometer (NanoDrop Technologies)^9^ was used to determine the concentration of the protein. After the concentration of the concentrated solution of each treatment was adjusted to the same level, a 20 μl crude enzyme solution was added to 5 μl 5x SDS loading buffer (50% 1-propanol, 20% Glycerin, 625 mM Tris/HCl, pH6.8, 10% SDS, 2% DTT and 0.2% Bromophenyl blue) and separated using sodium dodecyl sulfate-polyacrylamide gel electrophoresis (SDS–PAGE) in 12% gels. A Quantity One®1-D (1,709,600, Bio-Rad)^10^ was used to scan and analyze the result of SDS–PAGE. Each treatment was performed with three independent biological replicates.

According to previous methods, the crude PPO enzyme solution obtained was also used to assay the activity of the enzyme with the spectrophotometer method according to previous methods (Wang J. et al., 2016; Li H. et al., 2017). Taking 0.1 change of OD_420_ per minute as 1 PPO activity unit (U). The result was expressed as U mg^−1^ protein.

### Western Blot Analysis

*JrPPO1-*, *JrPPO2-*, and *JrGAPDH*-His6 fusion protein expressed in *E. coli* Rosetta-Gami 2 (DE3; Solarbio)^11^ cells were extracted. First, the crude extract was separated by SDS–PAGE, and then the recombinant protein strips were cut off and soaked in Tris-glycine buffer [25 mM Tris, pH 12.5, 250 mM glycine, and 0.5% SDS (w/v)] in dialysis bags (8–14 kDa; Solarbio).^11^ Finally, the purified protein (150 μg) of each target gene was used to produce the corresponding polyclonal antibody according to the method of the previous researcher (Li T. et al., 2017). The antibodies obtained were stored at −80°C until use.

Total protein was extracted from 0.5 g frozen hull sample (cv. Qingxiang) at 8 dpi using a buffer containing 220 mM Tris/HCl, pH7.4, 250 mM sucrose, 1 mM MgCl_2_, 50 mM KCl, and 10 mM β-mercaptoethanol. The molecular protein weights of *JrPPO1* and *JrPPO2* were calculated using DETAIBIO (DETAIBIO)^12^ 68.28 KDa and 67.73 KDa, respectively, and identified by using Prestained & Western Blot Marker (HaiGene).^13^ The extracted proteins were separated using SDS–PAGE in 12% gels and then subjected to Western blotting with antibodies against the *JrPPO1* and *JrPPO2* protein to detect corresponding walnut PPO protein accumulation as previously described (Li et al., 2020).

### Quantification of Photosynthetic Pigments and Microscopic Observation

About 0.5 g frozen sample was added into 80% acetone to extract photosynthetic pigments. A UV/VIS spectrophotometer (UV-3100, MAPADA)^14^ was used to determine chlorophyll a, chlorophyll b, and carotenoids levels by measuring the absorbencies at 663, 645, and 470 nm, respectively (Lu et al., 2012). Pigment contents were calculated as previously described (Lichthenthale, 1987). To observe chlorophyll fluorescence, the fruits at 8 dpi were cleaned using double-distilled water and absorbed the surface water with facial tissue in the zip lock bag. Then fruits were placed on ice. The tissue tray and blade were put on a cold table before cutting. The blade was used to cut the fruit hull longitudinally and to remove a tissue block with an area of 1 cm × 0.5 cm. The tissue block was placed on the pre-cooled tissue tray and embedded using an embedding agent (Tissue-Tek® O.C.T. Compound, Sakura® Finet).^15^ A freezing microtome (Cherry blossoms POLAR DM, Japan) was used to slice the embedded samples (8 μm thick). The slices were put on polylysine-treated slides, and inverted fluorescence microscopy (Leica DMi8, Germany) was used for visualization.

## Results

### VIGS-TRV System Induces Moderate Symptoms on Walnut (*Juglans regia* L.) Fruits

A pTRV-*JrPDS* recombinant was constructed (Figure 1A) with two 240-bp *JrPDS* fragments, each regulated by a double 35S promoter to verify whether the VIGS-TRV system can induce gene silencing in fresh walnut fruits. No photobleaching phenotype was observed in pTRV-00-infected fruit, though mild mosaic symptoms induced by the TRV itself emerged in comparison with Mock and Water control (Figure 1B). pTRV-*JrPDS-*infected fruits showed moderate photobleaching in the co-culture inoculation method (Figure 1B). Four inoculation methods were used to test the infectivity and efficacy of the VIGS-TRV system on the fruits of two cultivars, Xiluo2 and Qingxiang (Supplementary Table S1). Only the co-culture-treated fruits showed a symptomatic phenotype. The infection efficiencies at 10 dpi were 83 and 75% in “Xiluo2” and “Qingxiang,” respectively (Supplementary Table S1). No photobleaching phenotype was observed on fruits infected *via* injection (inoculation at equator and stalk; Baulcombe, 1999), friction (Rodriguesl et al., 2007), and vacuum infiltration (Maleksaeedi et al., 2014) until 20 dpi in both cultivars (Supplementary Figures S1, S2 and Supplementary Table S1). Co-culture was the only successful strategy for *J. regia* fruit infection since it showed a significant photobleaching phenotype. Therefore, the VIGS-TRV system can silence the endogenous *J. regia PDS* gene.

### Optimization of the VIGS-TRV Silencing System in *J. regia* Fruit

**Figure 2:**
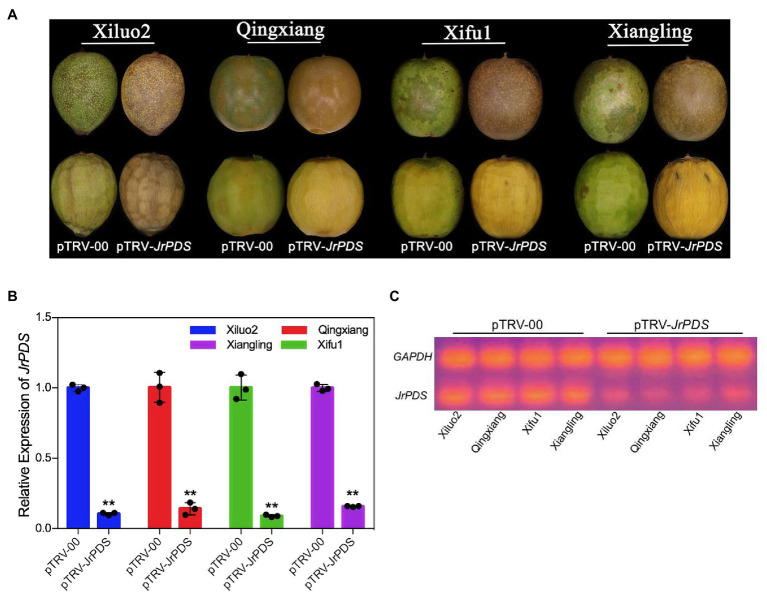
Schematic representation of the TRV genomes and the *JrPDS* silencing in *J. regia* fruits at 10 dpi. **(A)** *JrPDS* silencing in *J. regia* fruit hull. The fruits of four *J. regia* cultivars (cv. Xiluo2, Qingxiang, Xifu1, and Xiangling) were inoculated *in vitro*. The image shows the phenotype of fruits infected with pTRV-00 and pTRV-*JrPDS*. **(B)** The relative expression levels of *JrPDS* in pTRV-*JrPDS*-infected walnut fruits at 10 dpi. pTRV-00-infected fruits were used as control, and the mean of three biological repeats was calculated for individual assays. Bars indicate SD, and asterisks indicate statistically significant differences relative to the control values (Student’s *t*-test; ^**^p ≤ 0.01). **(C)** RT-PCR detecting *JrPDS* gene expression.

The infection characteristics and VIGS phenotype obtained under various treatments and conditions of co-culture were recorded to establish an optimal method for efficient and stable gene silencing in *J. regia* fruit (Table 1). Photobleaching phenotype was observed on the surface and the mesocarp of *J. regia* hull infected with pTRV-*JrPDS* at 10 dpi (Figure 2A). RT-PCR and qRT-PCR analyses revealed a substantial reduction in *JrPDS* transcript levels in the mesocarps of the four cultivars (Figures 2B,C), confirming that *JrPDS* silencing caused the photobleaching phenotype. Transcript level reduction of *JrPDS* was highest in “Xifu1” (90% of the control) with no significant difference between the four cultivars. Therefore, the VIGS-TRV system can induce gene silencing in all four *J. regia* cultivars.

**Table 1:** *JrPDS* silencing efficiency in cv. Qingxiang fruits inoculated with pTRV-*JrPDS* at different co-culture conditions with mature fruits.

Co-culture conditions^a^	Symptom appearance (dpi)^b^	Complete photobleaching (dpi)	Fruits obtained/Inoculated fruits^c^	Infection efficiency (%)^d^
Hyperosmotic pretreatment	2-3	8-10	31/54	85
No pretreatment	3-4	9-11	28/54	71
0 μM AS	2-3	8-10	29/54	80
10 μM AS	2-3	8-10	31/54	80
50 μM AS	2-3	8-10	32/54	81
100 μM AS	2-3	8-10	32/54	86
150 μM AS	2-3	8-10	33/54	86
OD_600_ of 0.1	2-3	8-10	30/54	80
OD_600_ of 0.2	2-3	8-10	30/54	56
OD_600_ of 0.3	1-2	-	6/54	10
OD_600_ of 0.5	-	-	0	0
OD_600_ of 1.0	-	-	0	0

a “Qingxiang” fruits were inoculated via the co-culture method. Before co-culture with *A. tumefaciens*, the fruits were plasmolyzed by soaking in 1/2 MS solution (pH 5.8) with 25% (w/v) sucrose for 5 h, except for the non-pretreated group.

b “-” The silencing phenotype did not appear.

c Fruit numbers obtained refer to the fruits invaded by *A. tumefaciens* carrying TRV. The ratio of fruits obtained to total fruit numbers inoculated indicated the inoculation success rate.

d The infection efficiency was the percentage of TRV-infected fruits to fruits with the *A. tumefaciens* invaded after inoculation. Visible photobleaching phenotype indicated the TRV infection in fruit.

The culture conditions of the co-culture inoculation were varied and optimized to improve the silencing efficiency of the VIGS-TRV system. The infection efficiency of hyperosmotic pretreatment was 14% higher than the no hyperosmotic pretreatment, with an earlier phenotype appearance. Besides, AS concentration did not affect (Table 1). The highest infection efficiency with complete photobleaching phenotype was obtained at an OD_600_ of 0.1 (Figure 2A; Table 1). Meanwhile, no infection occurred at an OD_600_ of 0.5 and 1.0 resulting in a dark brown phenotype (Supplementary Figure S3), indicating that high concentrations of the bacterial solution might cause anoxic death of the fruit.

### *JrPDS* Silencing Causes a Persistent Photobleaching Phenotype

**Figure 3:**
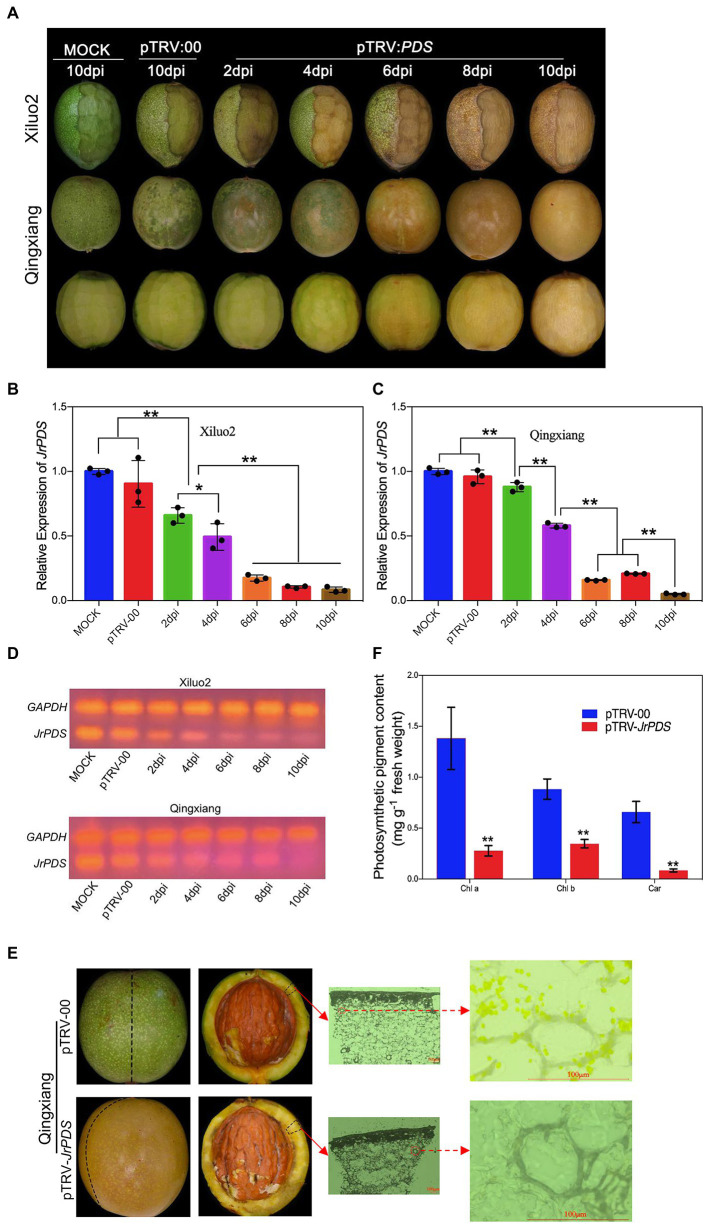
*JrPDS* silencing in the fruits of cv. Qingxiang and Xiluo2 using the VIGS-TRV system generates a persistent photobleaching phenotype. **(A)** Fruit phenotype of cv. Qingxiang and Xiluo2 infected with Mock control, pTRV-00, and pTRV-*JrPDS*. The fruits infected with Mock and pTRV-00 were photographed at 10 dpi, while those infected with pTRV-*JrPDS* were collected and photographed every 2 days. A quarter of the outer hull was peeled for “Xiluo2” before inoculation. FIGURE 3“Qingxiang” fruits were not peeled before inoculation, and their phenotypes at each sampling day were shown in both unpeeled and peeled states. **(B,C)** The significantly reduced *JrPDS* expression levels in silenced fruits relative to the Mock and pTRV-00. Data were pooled across experiments and analyzed using Student’s *t*-test. Values were expressed as means ± SD. The asterisks indicate statistically significant differences compared with the control at ^*^p ≤ 0.05 and ^**^p ≤ 0.01. **(D)** RT-PCR detecting low *JrPDS* expression in pTRV-*JrPDS*-infected fruits. **(E)** Microscopic observation on pTRV-00 and pTRV*-JrPDS*-silencing fruits at 8 dpi. Scale bar = 100 μm. **(F)** The significantly decreased photosynthetic pigments in *JrPDS*-silenced fruits, compared with pTRV-00-infected fruits. Each experiment was performed with three biological replicates, and samples were collected at 8 dpi. Values were expressed as means ± SD. Asterisks indicate a statistically significant difference compared with the pTRV-00 treatment (Student’s *t*-test; *p* < 0.01).

**Figure 4:**
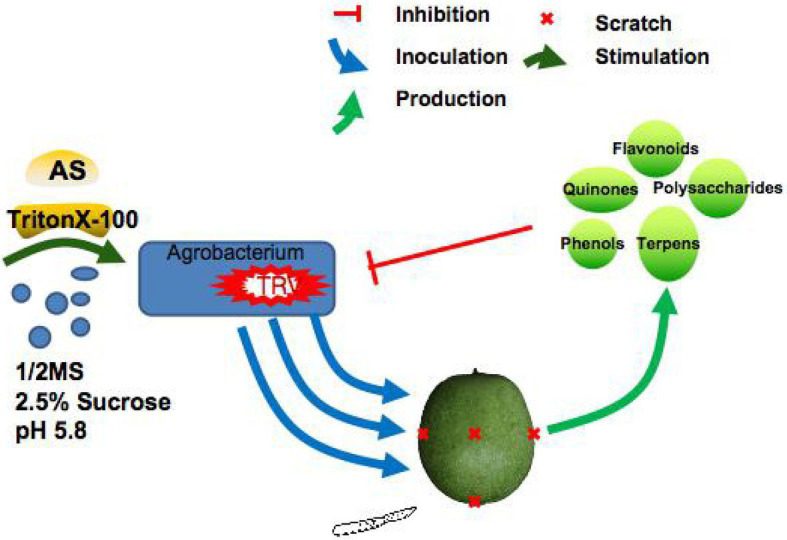
A model of TRV-mediated VIGS system for inoculating *J. regia* fruits *via* co-culture was constructed. The co-culture media had 1/2MS liquid medium with 2.5% sucrose, 100 μM acetosyringone (AS), 75 μM TritonX-100, and 2.5% sucrose. In this media, the *A. tumefaciens* with TRV showed sustained growth, which effectively mitigated the adverse effect of low infection rate caused by the death or poor growth of *A. tumefaciens*. In addition, the efficient replication of TRV significantly improved the TRV gene silencing efficiency: VIGS-TRV system in walnut fruits. TRV, tobacco rattle virus; VIGS, virus-induced gene silencing.

The *J. regia* fruit hulls infected with a silencing vector with a *JrPDS* fragment showed a very partial photobleaching phenotype from 2 dpi, and the incidence spread to the whole fruits at 8–10 dpi. The pTRV-00-infected fruit hull and Mock did not show the photobleaching phenotype until 10 dpi (Figure 3A). Peeling a quarter of the outer hull of “Xiluo2” before inoculation, an apparent photobleaching phenotype on the peeled part was observed at 2–4 dpi. In contrast, the phenotype was little on unpeeled parts, indicating that wounding promoted the infection. The photobleaching phenotype also appeared on the fruits of “Qingxiang” at 2–4 dpi, whereas their mesocarps showed no change after the fruits were peeled. The phenotype on both cultivars developed day by day, indicating that the infection initiated from the surface and spread gradually into mesocarps, as shown in Figure 3A. All these findings revealed that the optimized co-culture inoculation of *Agrobacterium*-mediated infection could lead to a complete photobleaching phenotype on *J. regia* fruit, which takes more than 8 dpi. Moreover, the large-scale shock on *J. regia* fruit hull tissue induced various substances from the fruit into the culture medium. The culture medium then darkened and developed mildew, and the fruits rotted (Supplementary Figure S3). Therefore, the mild trauma model (Figure 4) was adopted in subsequent experiments.

Quantitative RT-PCR analysis demonstrated that *JrPDS* expression in “Xiluo2” and “Qingxiang” challenged with pTRV-*JrPDS* decreased 88 and 78% compared with those challenged with the pTRV-00 control at 8 dpi, respectively (Figures 3B,C). The result verified the silence of *JrPDS* was successful and large scale occurred at 4 dpi, revealing the consistent photobleaching phenotype trend (Figure 3D). The chlorophyll and carotenoid contents of pTRV-*JrPDS*-infected fruit hull were significantly decreased (82, 57, and 90% reduction in chlorophyll a, chlorophyll b, and carotenoids, respectively; Figure 3F). The spontaneous chloroplast fluorescence was not observed in the pTRV-*JrPDS*-infected walnut hull, while the green fluorescence was observed in the control fruit hulls (Figure 3E). These findings collectively indicate that the albino phenotype can persist for a long time.

### *JrPPOs* Silencing in *J. regia* Fruits Hull

**Figure 5:**
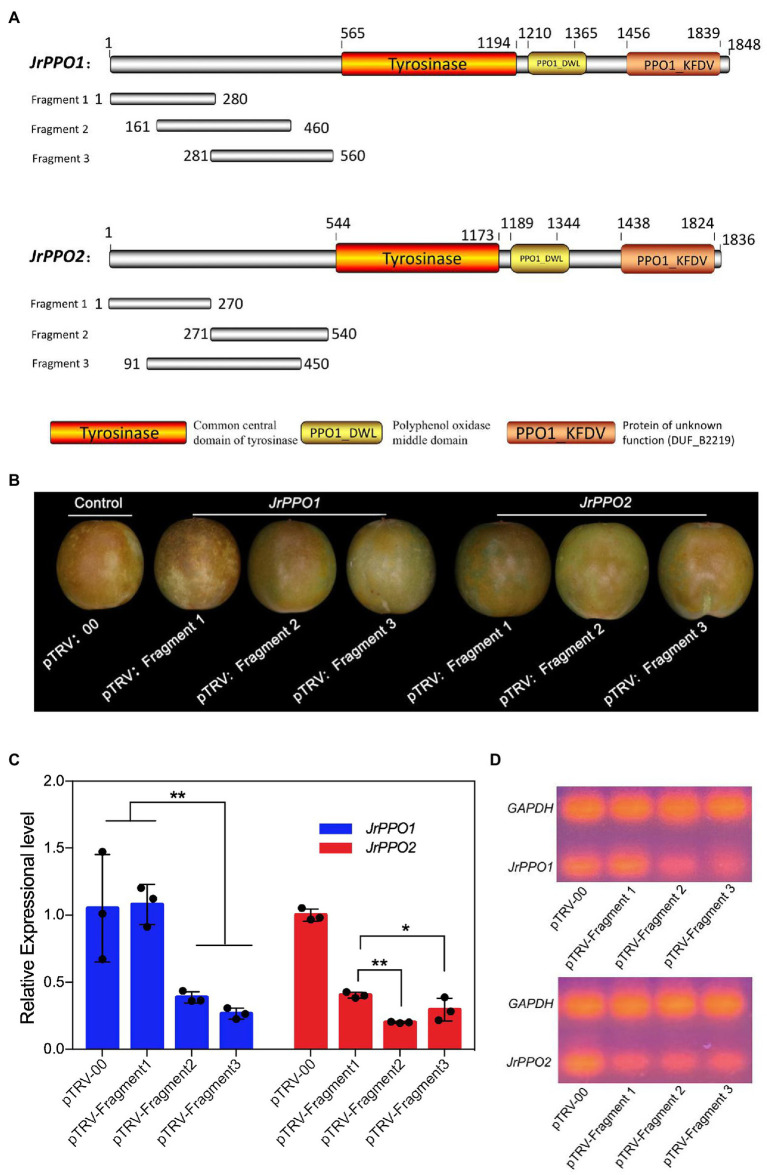
Schematic representation of *JrPPOs* fragments designing and screening. **(A)** *JrPPOs* silencing fragments design. The three fragments located in the non-conserved domain of *JrPPOs* were selected to prevent silencing interference. Colored boxes indicate the three conservative domains. **(B)** Pictorial representation of fruits treated with constructs harboring different fragments at 8 dpi. These fruits were photographed 1 h after the fruits were removed from the inoculation solution. The obvious difference in the phenotype of different experiments did not show until 1 h later. **(C)** Quantitative RT-PCR showing the decreased *JrPPOs* levels. The screening for the most efficient fragment was successful. Data were pooled across experiments, and Student’s *t*-test was used for analysis. FIGURE 5Values were expressed as means ± SD. The asterisks indicate statistically significant differences relative to the control at ^*^p ≤ 0.05 and ^**^p ≤ 0.01. **(D)** RT-PCR detecting the expression of the designed *JrPPO* gene silencing fragments.

Three gene fragments of *JrPPO1* and *JrPPO2* were amplified from the non-conserved domain of the corresponding target gene to separately clone into pTRV2 to increase the specificity of gene silencing and screening the most effective fragment (Figure 5A). pTRV-00-infected *J. regia* fruits showed the highest browning phenotype at 8 dpi, while those infected with TRV harboring *JrPPOs* fragments showed lighter browning. For the *JrPPO1* group, fruits infected with pTRV-fragment1 (*JrPPO1*) showed the same degree of browning as the control (Figure 5B). Fruits infected with pTRV-fragment3 (*JrPPO1*) showed the lightest browning symptom, and those infected with pTRV-fragment2 (*JrPPO1*) presented moderate browning symptoms. For the *JrPPO2* group, fruits infected with pTRV-fragment1 (*JrPPO2*), pTRV-fragment3 (*JrPPO2*), and pTRV-fragment2 (*JrPPO2*) showed the highest, moderate, and the least browning symptom, respectively (Figure 5B). Further, the results obtained from the qRT-PCR test indicated that the lower *JrPPOs* transcript levels corresponding to the less browning phenotype (Figures 5B,C). RT-PCR analysis of transcript level changes in each silencing treatment further confirmed the results of Quantitative RT-PCR (Figure 5D). Thus, fragment3-*JrPPO1* and fragment2-*JrPPO2* are the most suitable inserts for maximal *JrPPOs* silencing.

### The Biological Function Evaluation of *JrPPOs* in Delaying Fruit Browning Using VIGS-TRV System

**Figure 6:**
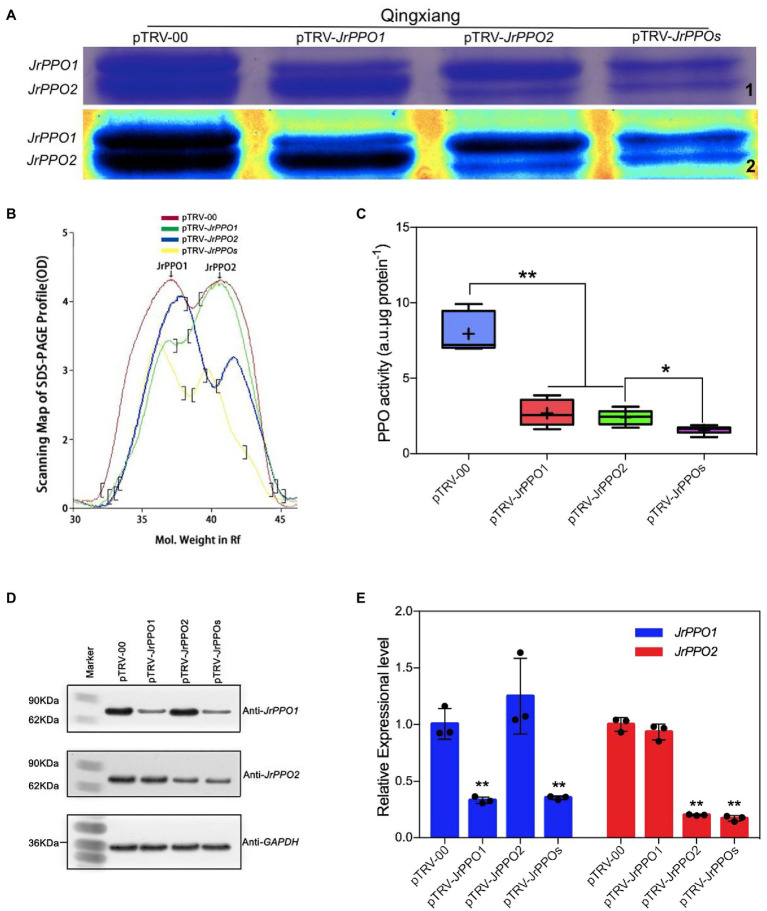
*JrPPOs* silencing using VIGS-TRV system alters protein content, gene transcription, and enzyme activity in fruits. Samples were collected from cv. Qingxiang infected with pTRV-00 or pTRV-*JrPPOs* at 8 dpi. **(A)** SDS-PAGE of *J. regia* Polyphenol oxidase protein in fruit hull. “1” indicates the native SDS-PAGE mode, “2” shows the standard spectrum mode with Quantity one-4.6.2. **(B)** The scanning profiles of the SDS-PAGE map used for comparison. **(C)** JrPPO activity assay in each sample after virus-induced gene silencing. The experiments were performed with three biological replicates. Student’s *t*-test was used for data analysis. Values were expressed as means ± SD. The asterisks indicate statistically significant differences compared with the control at ^**^p ≤ 0.01. **(D)** Western blot analysis of *JrPPO1* and *JrPPO2* protein accumulation in different treated fruits. The bottom panel shows western blot analysis of *GAPDH* protein from the same extracts, demonstrating equal protein loading. **(E)** Relative *JrPPO1* and *JrPPO2* gene expression in silenced and control fruit. Data analysis showed that the decrease of reverse transcription level was greater than the protein level showed **(B)** the consistent overall trend as described above. TRV, tobacco rattle virus; VIGS, virus-induced gene silencing. The asterisks indicate statistically significant differences relative to the control at *p ≤ 0.05 and **p ≤ 0.01.

The optimized VIGS-TRV silencing system and the most suitable insert fragments were used separately and simultaneously silence *JrPPOs* in cv. Qingxiang fruits (Jiang et al., 2017). SDS-PAGE showed that the PPO protein quantity significantly decreased in *JrPPO1*-, *JrPPO2*-, and *JrPPOs*-silenced fruits (Figure 6A). Scanning profiles of the SDS-PAGE gels showed that the JrPPO1 peak was significantly reduced (about 50%) in both *JrPPO1*- and *JrPPOs*-silenced fruits. The JrPPO2 peak was also reduced (about 35%) in both *JrPPO2*- and *JrPPOs*-silenced fruits (Figure 6B). No significant differences in the PPO activities were observed between fruits infected with pTRV-*JrPPO1* and pTRV-*JrPPO2*, while their PPO activities were significantly higher than the pTRV-*JrPPOs*-infected fruits (Figure 6C). Western blot hybridization showed that *JrPPO1* and *JrPPO2* protein accumulation were significantly different in various treated fruits (Figure 6D). qRT-PCR analysis indicated that *JrPPO1* transcript levels significantly decreased in the fruits infected with pTRV-*JrPPO1* and pTRV-*JrPPOs* (about 65–67%) compared with the pTRV-00-infected fruits. *JrPPO2* transcript levels also decreased in fruits infected with pTRV-*JrPPO2* and pTRV-*JrPPOs* (about 80–83%; Figure 6E). Taken together, the VIGS-TRV system using the co-culture inoculation method can be used for the rapid gene function analysis in *J. regia* fruits.

## Discussion

In the last few decades, global production of the Persian walnut (*J. regia* L.) has significantly increased due to the increased consumption rates. Therefore, researches on gene functions are helpful to improve the quality and biological characteristics by molecular breeding of walnut to meet the market demand better. Recently, walnut genome annotation assembled 32,498 gene models using two methods, including SOAPdenovo2 and MaSuRCA (Martínez-García et al., 2016). The rapid and simultaneous analysis of multiple genes in walnut is essential to study their function efficiently. However, *J. regia* tissue is unique. Therefore, limiting gene function analysis (Tang et al., 2000), especially the transgene function, mutants, or stable transgenic production in walnut is time costly and labor intensive with low efficiency. Thus, developing an easy and feasible approach needs a quick evaluation of the *J. regia* gene function.

VIGS technology has been considered one of the quickest approaches to evaluating the gene function (Ratcliff et al., 1997; Burch-Smith et al., 2004). At present, more than 40 viruses have been transformed into VIGS vectors, of which about 37 can be used for the gene silencing in dicotyledons (Gu et al., 2014; Liou et al., 2014), while only a few VIGS vectors are suitable for monocotyledons. BSMV and BMV are commonly used as VIGS tools for the gene function analysis in cereal plants, but their inoculation methods are highly dependent on gene gun (Meng et al., 2009), *in vitro* transcription friction inoculation (Holzberg et al., 2002; Scofield et al., 2005; Ding et al., 2006) or preincubation in intermediate host plants such as tobacco (Ding et al., 2007). While many viruses, including tobacco mosaic virus (TMV; Kumagai et al., 1995), potato virus X (PVX; Ruiz et al., 1998), and tomato golden mosaic virus (TGMV; Kjemtrup et al., 1998), are also effective vectors in VIGS, however, they share the same intrinsic disadvantage, i.e., high pathogenicity (Jones et al., 2001; Ratcliff et al., 2001). The VIGS phenotype is superimposed on and sometimes complicated by chlorosis, leaf distortion, and necrosis symptoms induced by virus infection. Furthermore, since these viruses cannot infect every cell, phenotypes caused by VIGS will be obscured in cells where the target gene is not silenced. Ratcliff et al. (2001) demonstrated that the TRV vector induces very mild symptoms, i.e., minimal pathogenicity, infects large areas of adjacent cells, and silences gene expression in growing points. Previous studies (Jiang et al., 2017; Li et al., 2019) have reported that the VIGS-TRV system is feasible for the genomic function study on fruits *via* visual phenotype evaluation. However, no studies have been reported regarding the application of VIGS technology in walnut tissues. This is the first study to successfully use TRV-based gene knockdown in walnut fruits for functional analysis of PPO-encoding genes (*JrPPOs*). The VIGS-TRV system was used to silence *JrPDS* and *JrPPOs*, with the subsequent development of their respective phenotypes during 10 d, implying the VIGS-TRV system can be used for the rapid gene function analysis in walnut fruits.

VIGS-TRV system’s silencing efficiency depends on the dynamic interaction between virus propagation and plant growth (Wang et al., 2013). Since *A. tumefaciens* mediate TRV to enter walnut cells and express TRV virus, factors influencing the invasion of *A. tumefaciens* into walnut tissue are crucial. This study also confirmed that different inoculation methods had various effects on the walnut gene silencing efficiency (Table 1; Supplementary Table S1). Due to walnut tissue being rich in phenolic substances (Stachel et al., 1985), the main anti-bacterial and anti-virus components, such as phenols, quinones, flavonoids, terpenes, and polysaccharides in *J. regia* fruits, can kill or strongly inhibit the *A. tumefaciens* with the TRV when the shock occurs on the surface of walnut fruits (Zhou et al., 2019), failing short-time incubation modes like injection, friction, or vacuum infiltration. During the co-culture process, *A. tumefaciens* grows and infects the fruits continuously, increasing the survival chances of *A. tumefaciens* carrying TRV, which agrees with the previous studies (Zheng et al., 2012). However, the present study found that the addition of AS did not make *A. tumefaciens* containing virus infecting walnut fruits easier (Zheng et al., 2012), which might be due to the specificity of walnut fruits. All the improvements on incubation led to the co-culture method showing the highest infection percentages (83% on cv. Xiluo2 fruits and 75% on cv. Qingxiang fruits; Supplementary Table S1). *PDS* is a phenotypic marker gene for VIGS vector development present in many plant species (Di Stilio et al., 2010; Ma et al., 2012; Pang et al., 2013). *JrPDS* silencing using the optimized VIGS-TRV system showed a persistent photobleaching phenotype that gradually spread and penetrated through all the fruit tissues (Figure 3A), rather than a transient local silencing phenotype (Jiang et al., 2017). However, whether the silencing system can be used in the subsequent experiments involving biotic or abiotic stress treatments as reported on other fruits (Ma et al., 2012; Kong et al., 2015; Ding et al., 2018) further studies.

In the study, the most suitable insert fragments resulting in the maximal and efficient silencing of the target gene-*JrPPOs* were successfully screened out using the optimized VIGS-TRV system. Surprisingly, the visual phenotype (Figure 5B) that resulted from the silencing of *JrPPO1* and *JrPPO2* was similar to that obtained from *JrPPOs* silencing (data not shown). The *JrPDS* silencing showed a brown phenotype, instead of the typical photobleaching phenotype, possibly due to the accumulation of phenolic substances and PPO in *J. regia* tissue, which could cause rapid phenol oxidation (Jariteh et al., 2011) in the peeled fruits. Figure 6 shows that *JrPPOs* mRNA levels, protein levels, and PPO activity decreased with the same trend, indicating the VIGS-TRV system can effectively knock down gene expression in walnut fruits. Given the growth environment of walnut tissue changes during co-culture, more studies are needed to evaluate whether other genes and their gene products can affect the results of this experiment.

Previous studies regarding Persian walnut (*J. regia*) mainly focused on the physiological aspects of walnut cultivation, its organic components, and their functional application (Cheniany et al., 2013; Jariteh et al., 2015; Jahanbani et al., 2016), and interaction of walnut-pathogen and resistance mechanisms of *J. regia* (Khodadadi et al., 2016, 2020). Rare studies on the molecular mechanism of post-harvest *J. regia* fruits have been reported. The rapid post-harvest browning in the hull triggers the subsequent deterioration of the kernel in fresh *J. regia* fruit. The *J. regia* PPO levels are directly associated with the quality of *J. regia* storage (Wang et al., 2017). Therefore, an efficient and rapid TRV-based silencing system can facilitate gene identification for agronomical control, promoting targeted molecular breeding and improving the walnut fruit quality. To our knowledge, this is the first study on gene expression knockdown in *J. regia* fruits using recombinant TRV. Therefore, this study can provide a basis for subsequent research on *J. regia* gene function analysis and improvement.

## Conclusion

TRV-based VIGS can knock down endogenous marker gene *JrPDS* accompanied by a substantial photobleaching phenotype in walnut (*J. regia* L.) fruits. With the optimized co-culture inoculation method, the knockdown of *JrPPOs* by VIGS-TRV was confirmed to be successful by anti-browning phenotype. Furthermore, it significantly decreased transcript and protein levels of the target genes. Therefore, the VIGS-TRV system was first established and adapted for the rapid function analysis of browning-related genes in *J. regia* fruits. Furthermore, it shows the potential application for other functional genomics studies on *J. regia*. Further, a model of the TRV-mediated VIGS system for inoculating *J. regia* fruits *via* co-culture was developed (Figure 4).

## Data Availability Statement

The original contributions presented in the study are included in the article/Supplementary Material, further inquiries can be directed to the corresponding author.

## Author Contributions

HM and YW designed and initiated this project and wrote the original manuscript. YW and NH performed the experiments. YW, NY, LQ, and YL analyzed the data. All authors have read and agreed to the final version of the manuscript.

### Conflict of Interest

The authors declare that the research was conducted in the absence of any commercial or financial relationships that could be construed as a potential conflict of interest.

## Footnotes

^1^http://www.omegabiotek.com.cn/

^2^https://www.ncbi.nlm.nih.gov/Structure/cdd/wrpsb.cgi

^3^https://hardwoodgenomics.org

^4^http://www.vazyme.com

^5^https://www.takarabiomed.com.cn/

^6^https://agbio.com.cn/

^7^http://www.thermofisher.com

^8^https://www.merckmillipore.com/CN/

^9^https://www.thermofisher.com/cn/

^10^https://www.bio-rad.com

^11^http://www.solarbio.com

^12^http://www.detaibio.com/sms2/protein_mw.html

^13^http://www.haigene.cn/html/show-274.html

^14^http://www.mapada.com.cn

^15^https://us.vwr.com/store/product/4692300/tissue-tek-o-c-t-compound-sakura-finetek
