# Supplementary material for: An Efficient Virus-Induced Gene Silencing System for Functional Genomics Research in Walnut (Juglans regia L.) Fruits
Source: Front Plant Sci. 2021 Jun 23;12:661633. doi: 10.3389/fpls.2021.661633 (PMC8261060; doi:10.3389/fpls.2021.661633)
Supplement: Supplementary file 1 [file Data_Sheet_1.PDF]

Supplementary data

Figure S1 | No photobleaching phenotype was induced by inoculating pTRV-*JrPDS* in the fruit of *J. regia* cv. Qingxiang.

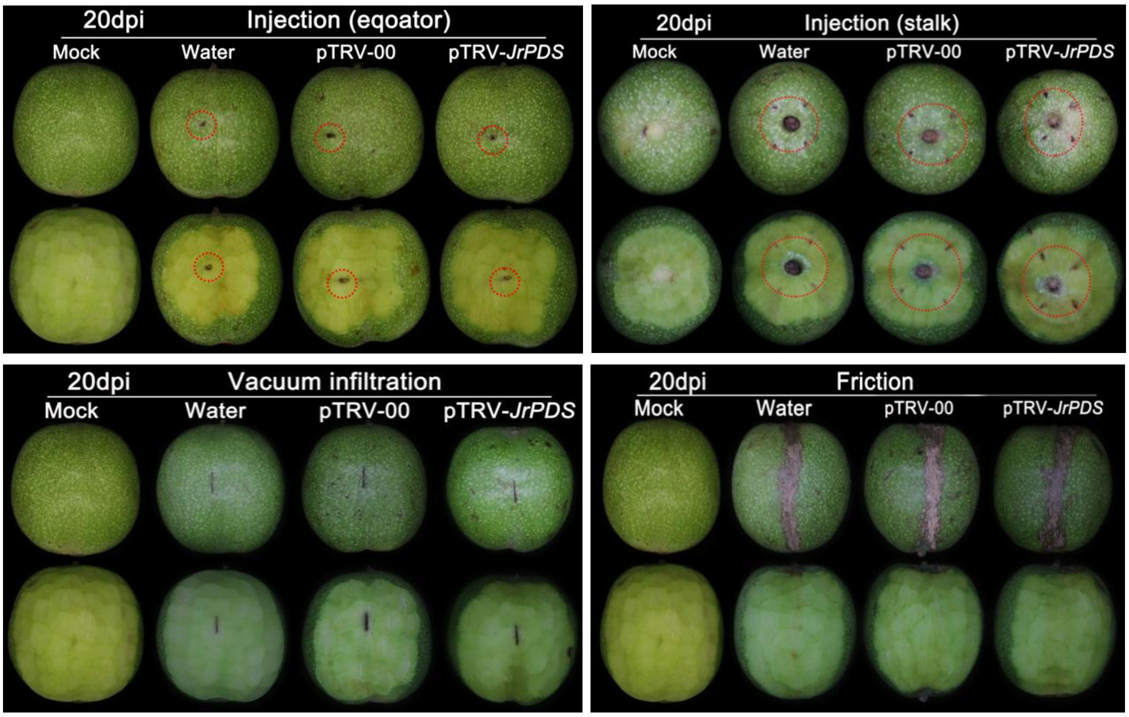

Figure S2 | No photobleaching phenotype was induced by inoculating pTRV-*JrPDS* in the fruit of *J. regia* cv. Xiluo2.

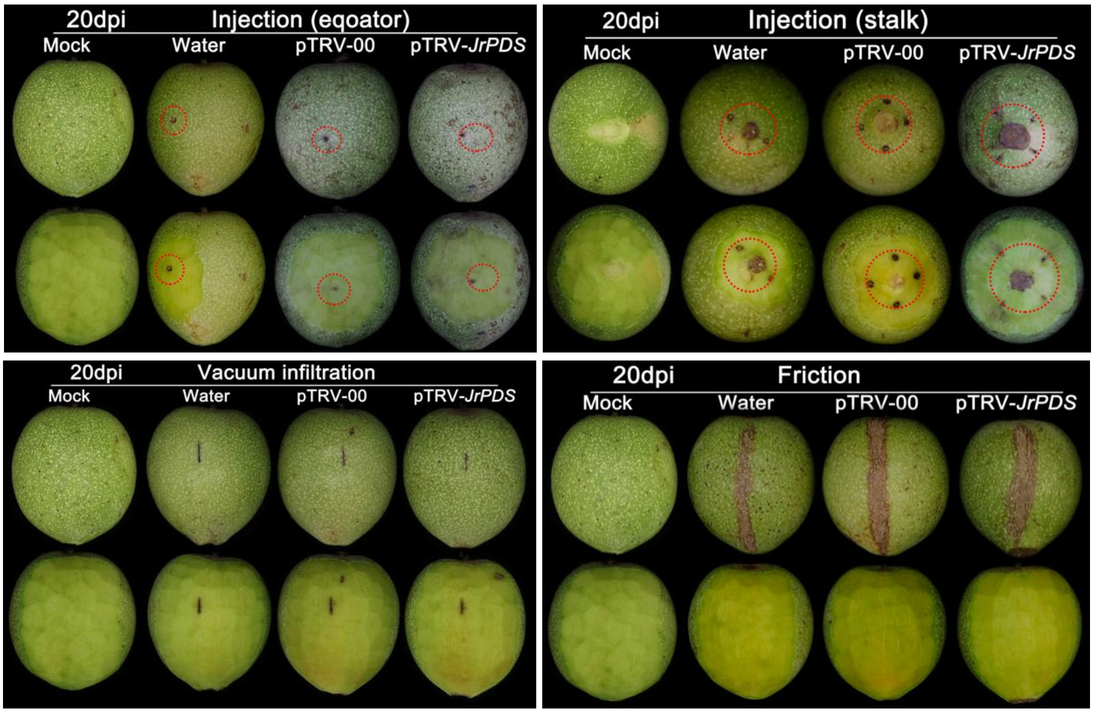

Figure S3 | *JrPDS* silencing phenotype induced by inoculating pTRV-*JrPDS* with co-culture at OD_{600} of 0.5 and 1.0.

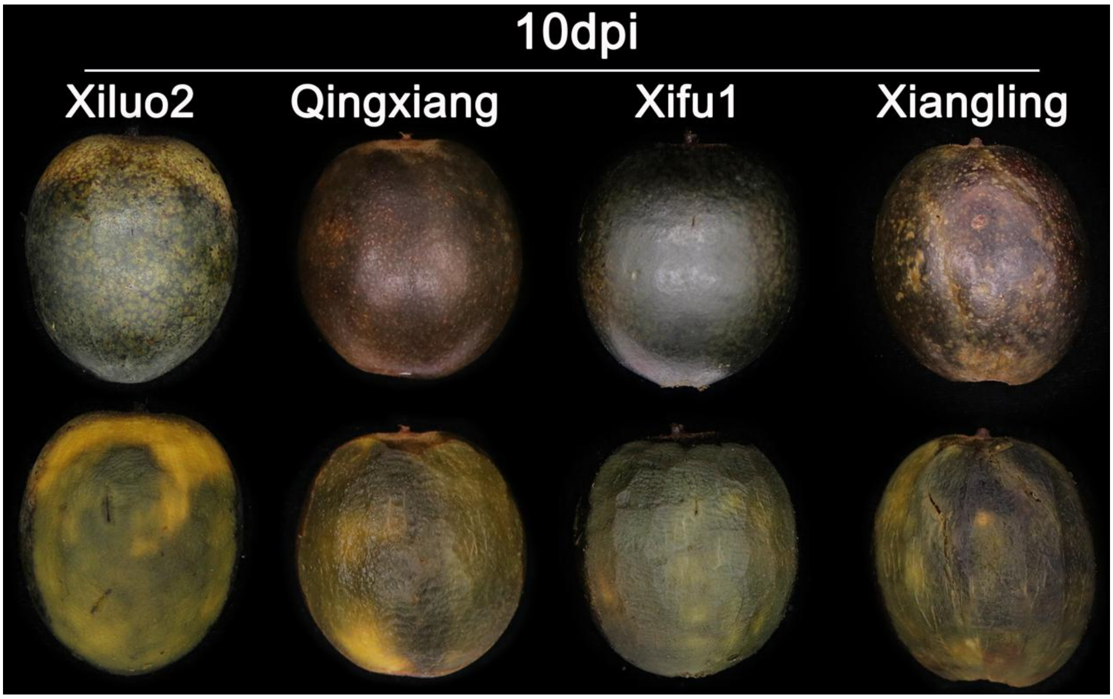

Table S1 | *JrPDS* silencing efficiency in the fruits of *J. regia* cv. Qingxiang and Xiluo2 by inoculating with different inoculation methods and inoculation regions.

Cultivar	Inoculation method[a]	Inoculation location[b]	Symptom appearance(dpi)[c]	Complete photobleaching (dpi)	Fruits obtained/inoculated fruits[d]	Infection efficiency (%)[e]
Xiluo2	Vacuum infiltration	Whole fruit	–	–	0/54	0
	Injection	Fruit handle	–	–	0/54	0
		Central fruit	–	–	0/54	0
	Friction	Fruit handle	–	–	0/54	0
		Central fruit	–	–	0/54	0
	Co culture	Whole fruit	2–3	8–10	36/54	83.33
Qingxiang	Vacuum infiltration	Whole fruit	–	–	0/54	0
	Injection	Fruit handle	–	–	0/54	0
		Central fruit	–	–	0/54	0
	Friction	Fruit handle	–	–	0/54	0
		Central fruit	–	–	0/54	0
	Co culture	Whole fruit	2–3	8–10	48/54	75

Table S2 | List of primers used for the construction of gene fragments for insertion into pTRV2.

Primer name	Sequence (5'-3')	
For silencing *JrPDS* (GenBank accession number: XM_018972693.1)		
P1	agaaggcctccatggggatccGAAAATCACAGTTTGGAAACTGCC	primer pairs for *JrPDS-fragment*
P2	gggacatgcccgggcctcgagGGAAGAGAGGTATGCGGCTTC	
For silencing *JrPPO1* (GenBank accession number: ACN86310.1)		
P5(Fragment1-F)	agaaggcctccatggggatccATGCTTTACCTTCACAGCTCACAC	primer pairs for *JrPPO1-fragments*
P6(Fragment1-R)	cgggacatgcccgggcctcgagCAAGGAGTACATCTCTTCTATCGAACTT	
P13(Fragment2-F)	agaaggcctccatggggatccGAAAGCAATACCACCACCTTACTACT	
P14(Fragment2-R)	cgggacatgcccgggcctcgagTTTGGCTAGGTAAAACAAAGTCTTTG	
P15(Fragment3-F)	agaaggcctccatggggatccGCCTCGGAGGCATGTATGG	
P16(Fragment3-R)	cgggacatgcccgggcctcgagTCAGCCGGGAGGGACTTC	
For silencing *JrPPO2* (GenBank accession number: XP_018805282.1)		
P9(Fragment1-F)	agaaggcctccatggggatccCTCTATGGTGTTGCTGGTCTTTACA	primer pairs for *JrPPO2-fragments*
P10(Fragment1-R)	cgggacatgcccgggcctcgagGTCAGCTGGAAGGGCTTTCA	
P17(Fragment2-F)	agaaggcctccatggggatccATGGCTTCTCTCTCAACTCAACCA	
P18(Fragment2-R)	cgggacatgcccgggcctcgagACCTCCTAGGCCAATGAGGACA	
P19(Fragment3-F)	agaaggcctccatggggatccCCAACCTTTCCAAAAAAGACCC	
P20(Fragment3-R)	cgggacatgcccgggcctcgagCAAGGGGGAGTTCTTGGTAGG	

For testing transformants		
UP-F	GGACATTGTTACTCAAGGAAGC	universal primer
UP-R	TAAATTACAAAAGACTTACCGATC	pairs for pTRV2

Table S3 | Primers for RT-PCR and quantitative RT-PCR analyses.

Primer name	Sequence (5'-3')	
P3	GCGATGCTTGGTGGACAG	For qRT-PCR detecting
P4	TTAGGGTTAATGAAGTTTAGTGCC	*JrPDS*
P7	CACCACGACCATTCGCACCC	For qRT-PCR detecting
P8	TTCCCTTGAGGATTTTGTCCATTAT	*JrPPO1*
P11	ACATTCCCCACGGTCCTG	For qRT-PCR detecting
P12	TTGGAGTGGTGAGCGAAAA	*JrPPO2*
JrGAPDH-F	ATTTGGAATCGTTGAGGGTCTTATG	For qRT-PCR detecting
JrGAPDH-R	AATGATGTTGAAGGAAGCAGCAC	*GAPDH*

Supplementary data Legends

Figure S1 | No photobleaching phenotype was induced by inoculating pTRV-*JrPDS* in the fruit of *J. regia* cv. Qingxiang. The fruit were photographed at 20dpi. Among the three inoculation methods, the manner of injecting and friction at the equator and the stalk of fruit was adopted.

Figure S2 | No photobleaching phenotype was induced by inoculating pTRV-*JrPDS* in the fruit of *J. regia* cv.Xiluo2. The graphic information is as described in **Figure S1**.

Figure S3 | *JrPDS* silencing phenotype induced by inoculating pTRV-*JrPDS* with co-culture at OD_{600} of 0.5 and 1.0. The dark brown phenotype on the surface of *J. regia* fruit inoculated with pTRV-00 and pTRV-*JrPDS* was photographed at 10 dpi. Due to hypoxia, *J. regia* fruit inoculated with pTRV-00 and pTRV-*JrPDS* showed the same tissue death phenotype.

Table S1 | *JrPDS* silencing efficiency in the fruits of *J. regia* cv. Qingxiang and Xiluo2 by inoculating with different inoculation methods and inoculation regions.

[a] *J. regia* fruit were inoculated via four inoculation methods.

[b] *The inoculation regions were chosen at the handle, middle of fruit and the whole fruit.*

[c] "-" *The silencing phenotype in J. regia fruit initateddid not appear.*

[d] *Fruit numbers obtained refer to the fruit invaded by Agrobacterium tumefaciens carrying TRV. The ratio of fruit obtained to total fruit numbers inoculated indicated the success rate inoculation.*

[e] *The infection efficiency was the percentage of TRV-infected fruit to fruit with the Agrobacterium tumefaciens invaded after inoculation. Visible photobleaching phenotype indicated the TRV-infection in fruit.*

Table S2 | List of primers used for the construction of gene fragments for insertion into the silencing vector.

The lowercase letters of the base sequence in this diagram represent the homologous arms designed for constructing the recombinants.

Table S3 | Primers for RT-PCR and quantitative RT-PCR analyses
